# Confocal Microscopy Improves 3D Microdosimetry Applied to Nanoporation Experiments Targeting Endoplasmic Reticulum

**DOI:** 10.3389/fbioe.2020.552261

**Published:** 2020-09-22

**Authors:** Annalisa De Angelis, Agnese Denzi, Caterina Merla, Frank M. Andre, Lluis M. Mir, Francesca Apollonio, Micaela Liberti

**Affiliations:** ^1^Inter University Center for the Study of Electromagnetic Fields and Biological Systems (ICEmB) at Department of Electronic Engineering and Telecommunications (DIET), University of Rome “La Sapienza”, Rome, Italy; ^2^Center for Life Nano Science@Sapienza, Istituto Italiano di Tecnologia, Rome, Italy; ^3^National Italian Agency for Energy, New Technologies and Sustainable Economic Development – Department of Sustainability (ENEA, SSPT) – Division of Health Protection Technologies, Rome, Italy; ^4^Université Paris-Saclay, Institut Gustave Roussy, CNRS, Metabolic and Systemic Aspects of Oncogenesis, Villejuif, France

**Keywords:** confocal fluorescence microscopy, microdosimetry, electroporation, electropermeabilization, nanosecond pulses, nanoporation experiments, endoplasmic reticulum, realistically shaped cell models

## Abstract

In the last years, microdosimetric numerical models of cells including intracellular compartments have been proposed, aiming to investigate the poration induced by the application of nanosecond pulsed electric fields (nsPEFs). A limitation of such models was the extremely approximate cell and organelle shapes, leading to an incorrect estimation of the electric field or transmembrane potential distribution in the studied domain. In order to obtain a reliable model of *in vitro* experiments and a one-to-one comparison between experimental and simulated results, here, a realistic model of 12 human mesenchymal stem cells was built starting from their optical microscopy images where different cell compartments were highlighted. The microdosimetric analysis of the cells group was quantified in terms of electric field and transmembrane potentials (TMPs) induced by an externally applied 10-ns trapezoidal pulse with rise and fall times of 2 ns, with amplitudes ranging from 2 to 30 MV/m. The obtained results showed that the plasma and endoplasmic reticulum (ER) membrane of each cell respond in a different way to the same electric field amplitude, depending on differences in shape, size, and position of the single cell with respect to the applied electric field direction. Therefore, also the threshold for an efficient electroporation is highly different from cell to cell. This difference was quantitatively estimated through the cumulative distribution function of the pore density for the plasma and ER membrane of each cell, representing the probability that a certain percentage of membrane has reached a specific value of pore density. By comparing the dose-response curves resulted from the simulations and those from the experimental study of De Menorval et al. (2016), we found a very good matching of results for plasma and ER membrane when 2% of the porated area is considered sufficient for permeabilizing the membrane. This result is worth of noting as it highlights the possibility to effectively predict the behavior of a cell (or of a population of cells) exposed to nsPEFs. Therefore, the microdosimetric realistic model described here could represent a valid tool in setting up more efficient and controlled electroporation protocols.

## Introduction

**FIGURE 1:**
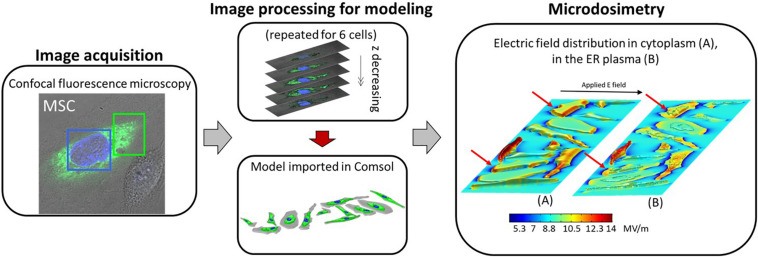
Block scheme of the microdosimetric modeling enhanced by confocal fluorescence microscopy, from the microscopy image acquisition to the microdosimetric analysis. The six reconstructed cells were doubled and randomly placed to form the cell mixtures. In the last block “Microdosimetry,” the electric field distribution over the entire domain for one of the four investigated cell mixtures, at the time corresponding to the end of the pulse plateau (*t* = 10 ns) is shown, highlighting the electric field induced in **(A)** the cytoplasm, and **(B)** the ER plasm.

Microdosimetry has gained a crucial role in the study of the electromagnetic field interaction with the biological matter. It offers the possibility to quantitatively evaluate local electromagnetic quantities induced by the application of an external electric field such as the electric fields local amplitude, the current densities and the local values of the induced transmembrane potential (TMP) differences. Such *in silico* precise descriptions allow a deeper understanding of the relationships between the applied electric field amplitudes and the biological effects experimentally observed (Apollonio et al., 2013; Merla et al., 2019). Moreover, following a scheme of infinite loop between “dry” and “wet” experiments (Kitano, 2002), used here as synonymous of *in silico* and *in vitro or in vivo* experiments, respectively, a microdosimetric model could represent a validation tool for the hypothesis formulated on the basis of the experimental observations (experiment-driven approach). Or, on the contrary, microdosimetry could provide predictions on the effects of the electromagnetic stimulation to be successively tested by *in vitro* and *in vivo* studies (model-driven approach). Indeed, the “infinite loop concept,” introduced by Kitano and further elaborated by the authors in Denzi et al. (2017; Figure 1), represents the possibility to get knowledge by a continuous exchange of information between numerical and experimental studies in which the outcomes from the first could be used as starting point of the second and *vice versa*. In the case of the experiment-driven approach as the former described above the need to reproduce real cellular and subcellular structure emerged as a crucial point, due to the dependence of the electric field induced in the cell on real irregular shapes (Denzi et al., 2016). Final purpose is to elucidate the physical and biological responses when relating the experimental exposure conditions to the observed effects.

Recently, different 2D numerical models of cell including intracellular compartments have been proposed in order to study the electroporation phenomenon induced by short pulsed electric fields on biological cells. These cell models were based on the transport lattice approach (Gowrishankar et al., 2006; Esser et al., 2010) or on meshed transport network (Smith et al., 2006; Zaklit et al., 2017). A limitation of such models relies in the shape approximation of the cell and organelles that can imply unreliability of the estimated spatial distribution of the electric field, of the transmembrane voltage, and of the pore density. Moreover, most of these models consider a single isolated cell that cannot be fully representative of a cell mixture, since, in the most of *in vitro* studies, researchers deal with populations of cells in suspension or in monolayers (Kotnik et al., 2010). Hence, in order to obtain a reliable model of *in vitro* experiments and a one-to-one comparison between experimental and microdosimetric results, it is necessary to build up an accurate model starting from the real geometrical and topological details of the cells used in the experiments (Pucihar et al., 2006, 2008; Towhidi et al., 2008; Kotnik et al., 2010). Indeed, it has been shown that the maximum TMP, computed for realistically shaped cells, can differ (by up to 30%) from models using simplified geometries (Pucihar et al., 2006). Towhidi et al. (2008), considered a population of irregularly shaped cells, and showed how the effects of local membrane curvature could lead to large variability (up to 100%) in the TMP determination. Further, it has been shown that it is harder to electroporate cells in a cluster due to the presence of multiple cell boundaries close each other and, in some cases, randomly oriented (Pavlin et al., 2002; Pucihar et al., 2007; Joshi et al., 2008; Kotnik et al., 2010). In Denzi et al. (2016) starting from microscopy images, we reconstructed a realistic 2D model of five human neuroblastoma cells with nucleus, considering their real shape, density and packaging, and applying pulses of different duration (100 μs, 60 ns, and 1 ns). In that study we confirmed the importance to take into account the real experimental conditions, and the duration (spectral content) of the applied pulse to understand the different responses. Indeed, generally speaking, microsecond electric pulses with amplitude of few kV/m (e.g., like those used in electrochemotherapy) are able to permeabilize the plasma membrane and, thus, can be used to favor the uptake of drugs and non-permeant molecules in the cell interior (Cadossi et al., 2014). nsPEFs with higher amplitude of the order of MV/m, due to their higher frequency content, are able to permeabilize also the subcellular membranes (such as mitochondria and ER) (De Menorval et al., 2016; Kulbacka, 2017). Poration of cellular and subcellular membranes has been widely used as a biotechnological tool for incorporating various molecules (genes, DNA, RNA, proteins, drugs, antibodies, and fluorescence probes) into many different kinds of cells (bacteria, yeast, plant, and mammalian cells). Hence, in the last years the study of the interaction between nanosecond electric pulses and cells with organelles like ER, have gained increasing interest. Some papers report experimental results showing that nanosecond pulses are able to induce intracellular calcium release from ER (Vernier et al., 2003; Joshi et al., 2007; Scarlett et al., 2009; Semenov et al., 2013; De Menorval et al., 2016). Calcium regulates a number of cell signaling, including cell apoptosis, enzyme activation, gene transcription, neurotransmitter release, and muscle contraction (Berridge et al., 2003; Sun et al., 2007; Frandsen et al., 2012; Petecchia et al., 2015). In particular, De Menorval et al. (2016) experimentally showed that a single pulse of 10 ns and few tens of MV/m is able to modulate the intracellular calcium release in a population of human mesenchymal stem cells (MSCs), by mimicking the spontaneous oscillations of Ca^2+^ ions in the cytosol. For a fully controlled modulation of the calcium release, it is important to exactly relate the gradient in Ca^2+^ concentration with the electric stimulation parameters (amplitude, duration and shape). This could be feasible using a model of the entire experimental poration set up as realistic as possible.

In the present work, we implemented a 3D model of the same cells used in the experimental work (De Menorval et al., 2016) reconstructed from cell images obtained with confocal microscopy. The aim is to demonstrate the importance of an accurate 3D realistic model of cells and subcellular organelles morphologies in relating the exposure condition to the electroporation phenomenon. Our novel model may be applied as a predictive tool for a better control of the experimental exposure settings.

First the method used to reconstruct the real shape of the cells will be described. Then, the implementation of the electromagnetic model will be detailed. Finally, a comparison between the experimental results and those from the simulation will be presented and discussed, in terms of the percentage of porated cells induced by increasing intensity of the applied electric field, with a major focus on the electric quantities that determine such effects.

## Materials and Methods

In order to reconstruct the realistic geometry representative of the real biological target we followed a procedure similar to the one of Denzi et al. (2016), Hanna et al. (2017). We employed the cell images provided by a confocal fluorescence microscope (Leica TCS SPE, Germany, 63×, 1.30 NA oil), that allows to visualize the different cell compartments treated with a specific fluorescent dye. Green dye (pcDNA-D1ER, excitation and emission at 480 and 535 nm, respectively) was used for the ER and a blue marker (Hoechst 33342, excitation and emission at 405 and 486 nm, respectively) for the cell nuclei. The images were taken without electronic zooming. Twenty-three slices were taken each time to produce stacks of cell images with a z distance of 0.8 μm between them. More details of the technique are reported in Hanna et al. (2017). Each slice recorded at different depths was processed as follows with a custom MATLAB^TM^ (v. 2016) automated routine: the RGB microscopy images were converted and normalized using double precision in order to improve the intensity contrast between the image pixels. Then, considering a color channel at a time, the converted images were elaborated through segmentation, clusterization (with the threshold values reported in Hanna et al., 2017) and edge extraction as detailed in Denzi et al. (2016) and shown in Supplementary Figure S1 of Supplementary. Finally, each region (cytoplasm, ER, and nucleus) was extruded for a thickness corresponding to the step of the microscopy procedure (z-stack of 0.8 μm), smoothed and harmonized. Then a STL file, in which all the information about the 3D model were stored, was extracted. The obtained 3D STL file of the cell can be directly imported in the used simulation software Comsol Multiphysics (v. 5.3). In Figure 1, a summary scheme of the used procedure and the resulted geometric model imported in Comsol Multiphysics are reported for six different mesenchymal stem cells (Hanna et al., 2017), then duplicated and placed in random position in order to obtain a mixture of 12 cells (an example is show in Figure 1 “Model imported in Comsol”). The procedure has been repeated four times in order to obtain four different mixtures of 12 cells. A more detailed scheme of the image processing and 3D cell reconstruction is reported in Supplementary Figure S1.

A numerical model was realized based on the dielectric properties of the cell as reported in Table 1 (Denzi et al., 2016). A parallelepiped box was used to represent the extracellular medium surrounding the cells. The right side of the box was set to the ground and the left one was excited by a single 10-ns trapezoidal pulse and variable amplitudes. The problem for the described geometry was solved in the Electric Currents mode of the AC/DC module of Comsol (Time Dependent Study). In this module, the voltage at each point is evaluated by the following equation:

**TABLE 1:** Electrical permittivity and conductivity of the cell compartments (Denzi et al., 2016).

Compartment	ε_*s*_	σ (S/m)
Membranes (Plasmatic and ER)	11.7	*s*(TMP)
Nucleus membrane	11.7	8.3 × 10^–5^
Plasmas	67	0.3
Extracellular medium	72	1.4

(1) $$-\nabla \left(\sigma_{i}\,\nabla V\right)-\varepsilon_{0}\,\varepsilon_{r}\,\nabla \left(\frac{\partial \left(\nabla V\right)}{\partial t}\right)=0$$

Where σ_*i*_ and e_*r*_ are the electrical conductivity and the relative permittivity of the specific compartment under study (extracellular medium, membranes, cytoplasm, ER plasm and nucleoplasm) and e_0_ is the permittivity of the vacuum and *t* the time. The gradient of the voltage ∇⁡*V* represents the TMP, the difference between the membrane internal and external voltage.

For all internal boundaries, continuity conditions were set. Moreover, in order to take into account the membrane conduction and the displacement currents, the *Contact Impedance condition:*

(2) $$n\cdot J_{1}=\frac{1}{d_{s}}\,\left(\sigma_{m}+\varepsilon_{0}\,\varepsilon_{m}\,\frac{d}{d\,t}\right)\,\left(V_{1}-V_{2}\right),$$

was imposed to the plasma, ER and nuclear membranes, instead of considering them as physical domains. This choice is justified by the fact that we were not interested here in the events occurring inside the membrane, avoiding the problem of generating a mesh of very small elements for the membrane interior (Pucihar et al., 2007, 2008). The pore formation dynamics were studied through the asymptotic model proposed by DeBruin and Krassowska (1999), implemented in the model using the Boundary ODEs (ordinary differential equations) and DAEs (differential-algebraic equations) application of the Mathematics module:

(3) $$\frac{d\,N}{d\,t}=\alpha \,e^{{\left(\frac{T\,M\,P}{V_{e\,p}}\right)}^{2}}\,\left(1-\frac{N}{N_{0}}\,e^{-q\,{\left(\frac{T\,M\,P}{V_{e\,p}}\right)}^{2}}\right)$$

N is the density of the pores forming per second and per m^2^ across the membrane. The other parameters are specified in Table 2. The pore creation determines an increase in the membrane conductivity, as further conducting pathways opened, increasing the current density flow across the membrane. For considering this event, the following formula was used:

**TABLE 2:** Parameters used in the asymptotic equation of the pore formation (DeBruin and Krassowska, 1999).

Parameter	Value	Description
σ_*m0*_	1.1 × 10^–7^ (S/m)	Initial conductivity
*r*_*p*_	0.76 (nm)	Pore radius
α	10^9^	Electroporation parameter
*q*	2.46	Electroporation constant
h	5 (nm)	Membrane thickness
*N*_*0*_	1.5 × 10^9^ (m^–2^)	Equilibrium pore density
*V*_*ep*_	258 (mV)	Characteristic voltage of electroporation

(4) $$\sigma_{m}=\sigma_{m\,0}+\sigma_{e\,p}$$

adding to the unporated membrane conductivity σ_*m*0_, the term σ_*ep*_ representing the changes in the conductivity of the membrane due to the pores formation, as in Mercadal et al. (2016).

(5) $$\sigma_{e\,p}=N\,\frac{2\,\pi \,r_{p}^{2}\,\sigma_{p}\,h}{\pi \,r_{p}+2\,h}$$

(6) $$\sigma_{p}=\frac{\sigma_{e}-\sigma_{i}}{l\,n\,\left(\frac{\sigma_{e}}{\sigma_{i}}\right)}$$

All the parameters not defined above are reported in Table 2.

In the present study, the same range of electric field used in De Menorval et al. (2016) from 0 to 36 MV/m was simulated. More specifically, a time domain study was performed simulating single trapezoidal 10-ns long pulses (2 ns rise and fall times) at 2.7, 6, 8.8, 10, 13, 16.2, 21, 25.2, 27, 36 MV/m, the same tested field amplitudes of the experimental study (De Menorval et al., 2016). Four different simulations have been performed, remixing the 12 cells in different positions and orientations with respect to the electric field direction, investigating a total of forty-eight cells.

## Results

In the last block of the schematic diagram in Figure 1 the electric field distribution over the entire domain for one of the four investigated cell mixtures is shown, at the time corresponding to the end of the pulse plateau (*t* = 10 ns). In panel (a), we report the extracellular electric field in 2D at a given quote together with the 3D representation of the induced electric field in the cytoplasm. In a similar way, in panel (b) we reported a 2D slice plot with the external and cytoplasmic electric field distribution together with the 3D electric field distribution on the ER plasma. The electric field penetrates the cells since the first instants of the pulse application and easily arises because of the displacement current associated with the distributed capacitance of the plasma membrane (Supplementary Figure S2). When the pulse is at the end of its plateau, the electric field within the cell is higher probably due to the pore conduction that becomes more important than passive displacement currents (Gowrishankar et al., 2006). Moreover, as the irregular shape of the plasma and ER membrane causes changes in the intensity and directions of the electric field similarly to what discussed in Denzi et al. (2016), the intracellular field is distributed non-uniformly. In some cells of the mixture, the electric field penetrates more easily than in others, in particular the cells exposing a larger area of membrane resulted in the higher amount of internal electric field. By comparing the duplicated cells (as those indicated by the red flashes in Figures 1A,B), one can notice that the internal field is very different. As the size and the shape are the same in this case, these results suggest that the position occupied by the cell in the chamber (i.e., the presence of the surrounding cells) and the orientation of the normal to the membranes with respect to the applied electric field direction, also affects the cell response/behavior. The external electric field experienced by each cell is strongly affected by the cell placement. Indeed, where the cells are more packed, the electric field presents local variations in terms of direction and intensity due to the interfering cells.

**FIGURE 2:**
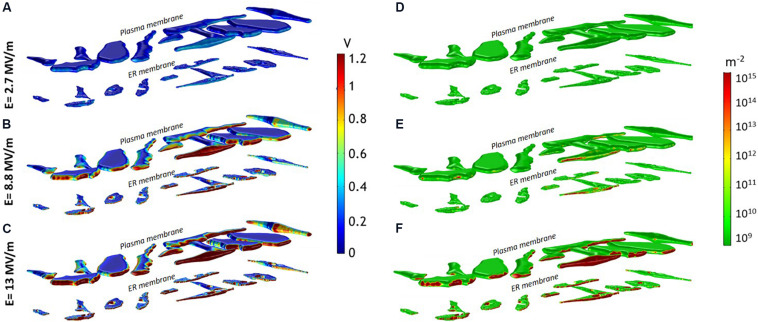
**(A–C)** Induced TMP and **(D–F)** pore density spatial distribution on the entire investigated domain (12 cells) at 10 ns for an applied pulse amplitude of **(A,D)** 2.7 MV/m, **(B,E)** 8.8 MV/m, **(C,F)** 13 MV/m.

This heterogeneity in the cell response well reflects the non-uniform TMP induction on the opposite sides of the membranes (Figures 2A–C). The TMP is generally considered a valid indicator of the electroporation occurrence. Experimental studies report different electroporation thresholds ranging from 0.2 and 1 V (Denzi et al., 2016), depending on cell type and experimental conditions (Merla et al., 2017). Here, a TMP > 1 V has been considered as the threshold value for the onset of the pore formation in the membranes. In Figures 2A–C the spatial map of the TMP induced at the end of the pulse plateau (*t* = 10 ns) by three increasing electric field intensities, (A) *E*_1_ = 2.7 MV/m, (B) *E*_2_ = 8.8 MV/m and (C) *E*_3_ = 13 MV/m is shown. For each field condition, the upper row reports the TMP on the plasma membrane and the lower row the TMP on the ER membrane. At 2.7 MV/m, no cell experienced a TMP >1 V (Figure 2A). At 8.8 MV/m, the red patches of the plasma and ER membranes are those where the TMP is above the threshold value of 1 V (Figure 2B). As evident, the induced TMP does not reproduce the typical symmetry of the regularly shaped cells, presenting strong local variations on the membrane of the same cell and from cell to cell. The extremely folded membrane experiences different TMP due to the fact that the cosine law in the Schwan’s equation (Foster and Schwan, 1986) loses its validity in irregular structures as reported also in Pucihar et al. (2006), Denzi et al. (2016) and Hanna et al. (2017). At 13 MV/m more extensive patches of plasma and ER membrane experienced a TMP >1 V in all the cells (Figure 2C).

In a more quantitative manner, we can derive the TMP trend during the stimulation period on the plasma and ER membranes of the same cell. The induced TMP averaged on the plasma membrane (Supplementary Figure S3) was higher than but very close to that on ER membranes for all the applied electric fields, confirming that a 10-ns electric pulse is potentially able to partially porate the ER with electric field values slightly greater than the ones needed to porate the plasma membrane. This result is clearly related to the high frequency content of the short pulse, mainly associated to the fast rise and fall times (2 ns).

In Figures 2D–F is reported the spatial distribution of the pore density over the same cell mixture and for the same three electric fields considered in Figures 2A–C. As evident from a qualitative inspection, some portions of the plasma and ER membranes showed a more important electroporation than others (significant pore density ≥ 10^14^). Indeed, in good accordance with the experimental results and with the simulated TMP maps, a single 10-ns pulse of 2.7 MV/m was insufficient to electroporate the plasma and the ER membranes in all the cells (Figure 2D). The plasma membrane of some more responsive cells starts to be porated at 6 MV/m (not shown), while for porating the ER membrane an electric field of at least 8.8 MV/m was necessary (Figure 2E), confirming that slightly lower amplitudes of nsPEF are needed to permeabilize the plasma membrane than those required for the ER membranes. At 13 MV/m both plasma and ER membranes of most of the cells were efficiently porated (Figure 2F). Interestingly, once the threshold for the ER electroporation is reached (at electric field > 9 MV/m), pore density could be even higher than that of the plasma membrane as showed in Supplementary Figure S4. Here, the maximum density of pores reached at each applied electric field intensity is reported for two selected cells, representative of the general cell behavior. This result is in accordance with the experimental observation that nsPEF <9 MV/m induced a modest peak of intracellular calcium, only due to the poration of the plasma membrane, while for amplitudes higher than 9 MV/m, when both plasma and ER membrane were permeabilized, a more important and sharp increase of the calcium concentration was recorded (De Menorval et al., 2016).

**FIGURE 3:**
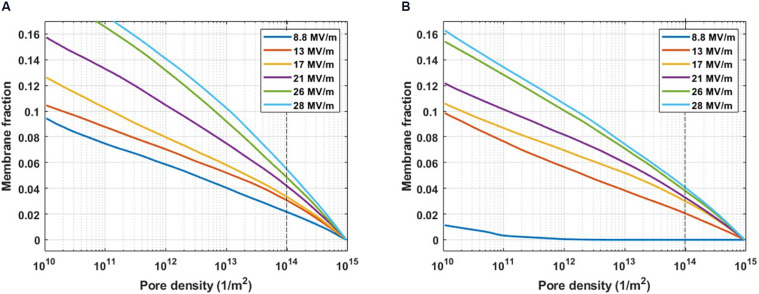
Pore density histogram on a single cell evaluated for the complementary cumulative distribution function. The fraction of the PM **(A)** and ER **(B)** membrane where the pore density reached at least the value indicated on the x-axis, at increasing electric field amplitudes from 8.8 to 28 MV/m. The dashed line indicates the threshold value for a significant density of the pore (10^14^ m^– 2^).

Supplementary Figure S5 reports an example of the cumulative distribution function directly estimated from the simulations, it represents the probability that a certain percentage of membrane has reached a value less than or equal to a specific pore density. The curves in Figure 3 were derived from the ones in Supplementary Figure S5 as their complementary cumulative distribution function (known also as reliability function; Ebeling, 2010).

**FIGURE 4:**
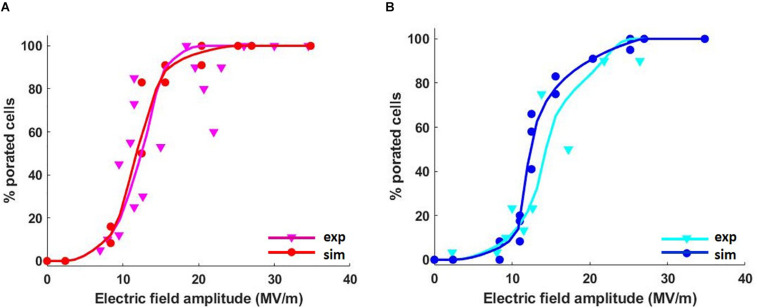
Average percentage of the cells with porated **(A)** plasma and **(B)** ER membranes in four different mixtures as a function of the applied electric field. The red and blue curves represent the average simulated data interpolation, while the magenta and the cyan curves are the interpolation of the average experimental data. Cubic Spline Interpolation was used for both the simulated and the experimental data.

Figure 3 represents the cumulative fraction of the plasma (Figure 3A) and ER (Figure 3B) membranes which has reached a density of the pores of at least the value reported in the x-axis, induced by increasing electric field amplitude (from 8.8 to 28 MV/m), at the end of the simulation (*t* = 10 ns). In particular, in the case of the cell reported in Figure 3, when an electric field of 8.8 MV/m is applied, a fraction of the plasma membrane equal to 0.02 is significantly porated (pore density ≥ 10^14^) (Figure 3A). In order to observe a comparable percentage in the ER membrane, a pulse amplitude of at least 13 MV/m becomes necessary (Figure 3B). Increasing amplitudes of the applied electric field result in increasing fractions of porated plasma and ER membranes patches, as expected. Then, the response of the cell mixture to the electric stimulation was quantified in terms of percentage of the porated cells in function of the applied electric field amplitudes. This percentage was evaluated, for each electric field intensity, as the ratio between the number of cells that, at the end of the stimulation (*t* = 12 ns), have reached the same fraction of porated membrane, according to Figure 3, and the total number of cells. The dose-response curves obtained considering different fractions of porated membrane, ranging from 0.01 to 0.05 overlapped to the experimental curve, are reported in Supplementary Figure S6. As in the experimental study where De Menorval et al. (2016) used two different extracellular conditions (in the presence and in the absence of external Ca^2+^), the poration of the plasma membranes (Supplementary Figure S6A) and the ER membranes (Supplementary Figure S6B) were investigated separately. A very good matching between the simulated and the experimental results was found when a fraction of the porated membrane of both the plasma and the ER equal to 0.02 is considered sufficient for permeabilizing the membrane (Figure 4). Here, the solid lines represent the average of the percentages resulted from the four simulations, reported as scattered points (small circles) in the same figure. A pulse amplitude of 7 MV/m is needed to efficiently porate the plasma membrane of about 10% of the cells in the mixture. 13 MV/m induced poration in 50% of the plasma membranes, while 17 MV/m is required to porate the plasma membrane in 100% of the cells (Figure 4A). Concerning the ER membranes poration (Figure 4B), an applied electric field of 9 MV/m was required for the onset of the poration (10% of the cells); while at 26 MV/m the ER membrane was extensively porated in 100% of the cells. These results, obtained in 3D realistic model, reconstructed from real images, are in good accordance with those obtained experimentally, investigating a number of cells (i.e., 48 in simulations vs. around the double in experiments).

## Discussion

nsPEFs induce the transient permeabilization of the cell membranes with an extent that depends on the nsPEF parameters (amplitude, duration, number, and repetition rate in case of a pulse train). However, the model has highlighted strong differences in the responses of each cell in the mixture when the same nsPEF was applied. This is because the position of the cell, the proximity of other cells as well as its orientation with respect to the electric field direction have an important impact on the cell response to the stimulation. As shown, not all the cells in the mixture experiment the same electric field amplitude, as the presence of other cells could mask the external field. In the part of the exposure chamber where the cells are more isolated, a higher electric field penetrates the cell and the ER. Indeed, due to the high frequency content of a 10-ns pulse with fast rise and fall times, the membrane becomes more conductive, allowing the subcellular electric field to fast increase. Another important aspect highlighted by the simulation concerns the possibility to estimate the total amount of the cellular and subcellular membranes experiencing an electric field able to porate them, and the degree of the poration. The general trend of a cell in the mixture reveals that a single 10-ns PEF is able to porate both the ER and plasma membrane with field intensities slightly different. In particular, the amplitude of the electric field necessary to porate the plasma membrane is moderately lower than that necessary to permeabilize the reticulum membrane as experimentally observed in Semenov et al. (2013), De Menorval et al. (2016).

Note-worthy, our realistic model is able to relate the percentage of the cellular and subcellular porated membrane to the applied electric field amplitude (and, presumably, to other PEF parameters like duration, rise and fall times, repetition rate). In our specific case, a fraction of 0.02 of porated membrane has resulted sufficient for permeabilizing the membrane at a detectable fluorescence intensity, proportional to the intracellular calcium concentration increase. Higher fractions of porated membrane would allow higher cytosolic calcium concentration. In that way, the presented model could be used to correctly set the PEF parameters for manipulating the calcium concentration on demand, and to control the related biological processes. Moreover, the extremely good accordance between the experimental curve of the cells presenting an observable calcium peak and the simulated dose-response curve when considering a porated membrane fraction of 0.02, suggests that this value could represent the minimum percentage of membrane that has to be porated in order to obtain an efficient effect in the experimental counterpart. Although we cannot completely replicate the experimental condition, like for example, the cell concentration in the pulsed sample, or their adhesion on the cover slip, the obtained results highlight the effectiveness of our numerical model in predicting the real response of a cell population to nsPEFs.

## Conclusion

A 3D realistic model of six human mesenchymal stem cells with ER and nucleus was built starting from their optical confocal microscopy images where different cell compartments were highlighted (Hanna et al., 2017). Confocal florescence microscopy has enabled the possibility to build such realistic 3D models from real images of 12 cells. Each reconstructed cell geometry was imported in Comsol Multiphysics (v. 5.3), duplicated and put in random positions as to obtain a mixture of 12 cells. The microdosimetric analysis of the group of cells was quantified in terms of electric field and TMPs induced by an externally applied 10-ns trapezoidal pulse with rise and fall times of 2 ns, with amplitudes ranging from 2 to 36 MV/m as to simulate the same electric field used in the experimental work of (De Menorval et al., 2016). Then, the cell-by-cell pore density estimation from the simulation was used to evaluate the percentage of electroporated cells as a function of the applied electric field intensity. The comparison between the simulated dose-response curves with those obtained experimentally by De Menorval et al. (2016) displayed a very good matching of results for plasma and ER membrane when a fraction of 0.02 of the porated membrane is considered sufficient for permeabilizing the membrane. The simulation has highlighted that plasma and ER membrane of each cell responds in a different way to the same electric field amplitude. As a consequence, also the threshold for an efficient electroporation is highly different from cell to cell. Indeed, some portions of the cell and ER membranes showed a more important electroporation than others, coherently with the TMP estimation, due to their extremely irregular and folded structure. The non-uniformity in the induced electric field distributions was due to the differences in shape, size, and position of each cell and its orientation with respect to the applied electric field direction. The relevance of this study lays in the possibility to effectively predict the behavior of a cell (or of a population of cells) exposed to a nsPEF with specific parameters. Therefore, the microdosimetric realistic 3D model described in this study, could represent a valid tool in setting up a more efficient and controlled electroporation protocol.

## Data Availability Statement

All datasets generated for this study are included in the article/Supplementary Material.

## Author Contributions

ADA performed the simulation, analyzed and elaborated the data, and wrote the manuscript. AD built the 3D model of the cell used in this study and reviewed the manuscript. CM contributed to the analysis and understanding of data and reviewed the manuscript. FMA and LM are the authors of the previous experimental work related to this article, provided the confocal microscopy images used for modeling the cells and reviewed the manuscript. FA and ML are the PI of this research study. All authors contributed to the article and approved the submitted version.

## Conflict of Interest

The authors declare that the research was conducted in the absence of any commercial or financial relationships that could be construed as a potential conflict of interest.
