# Supplementary material for: Confocal Microscopy Improves 3D Microdosimetry Applied to Nanoporation Experiments Targeting Endoplasmic Reticulum
Source: Front Bioeng Biotechnol. 2020 Sep 22;8:552261. doi: 10.3389/fbioe.2020.552261 (PMC7537786; doi:10.3389/fbioe.2020.552261)
Supplement: Supplementary file 1 [file Presentation_1.PPTX]

## Slide 1
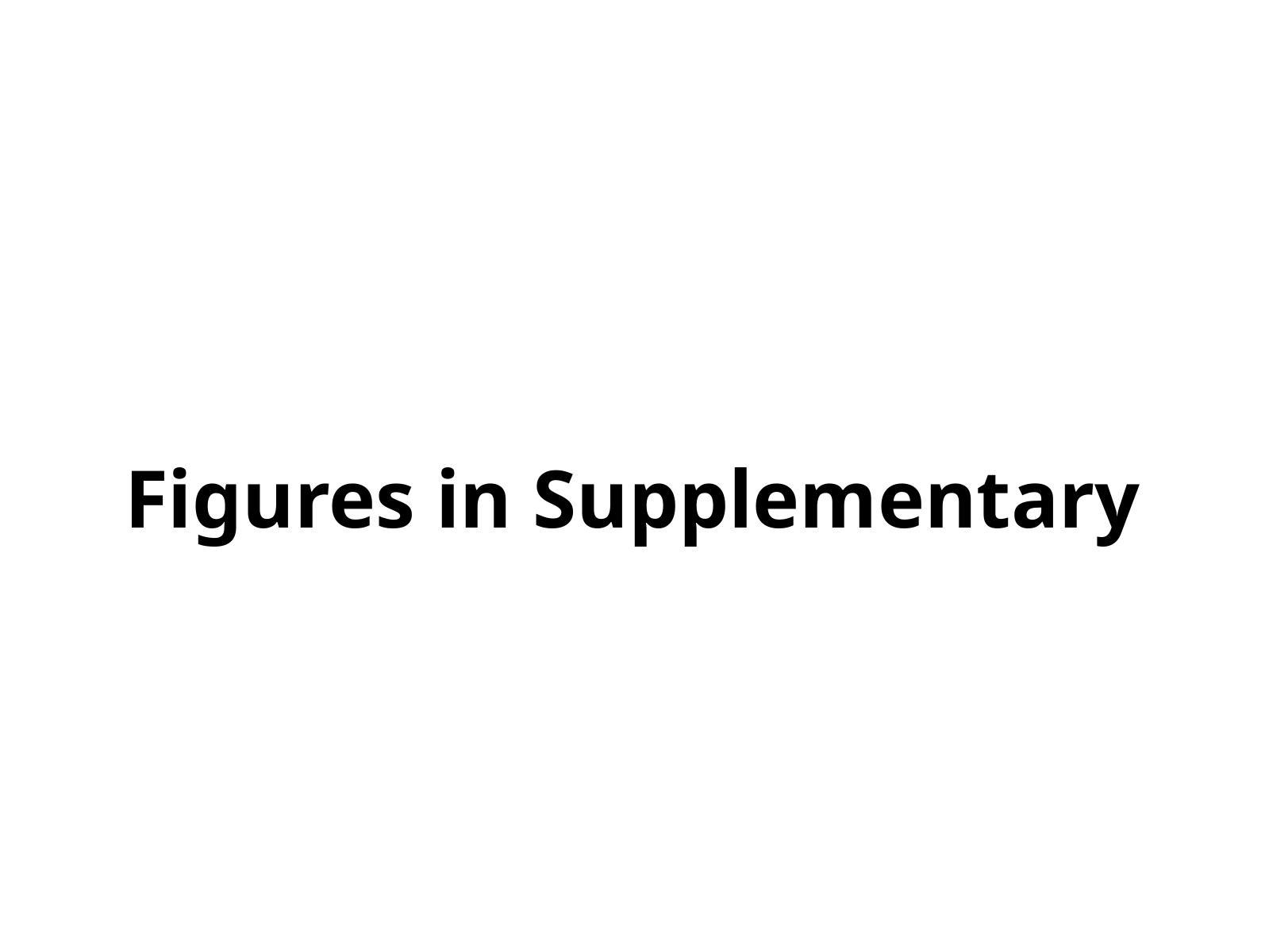

# Figures in Supplementary

## Slide 2
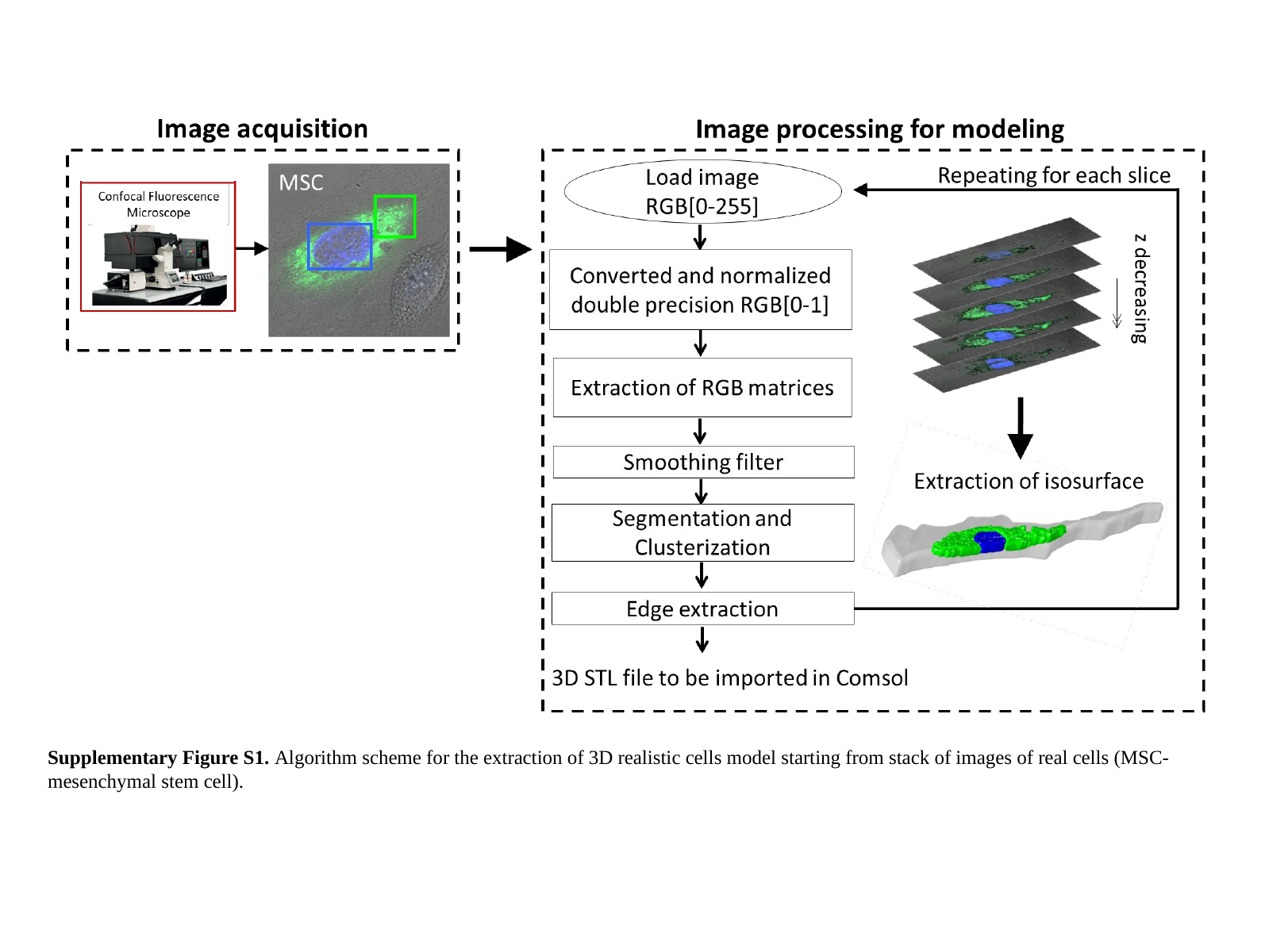

Supplementary Figure S1. Algorithm scheme for the extraction of 3D realistic cells model starting from stack of images of real cells (MSC-mesenchymal stem cell).

## Slide 3
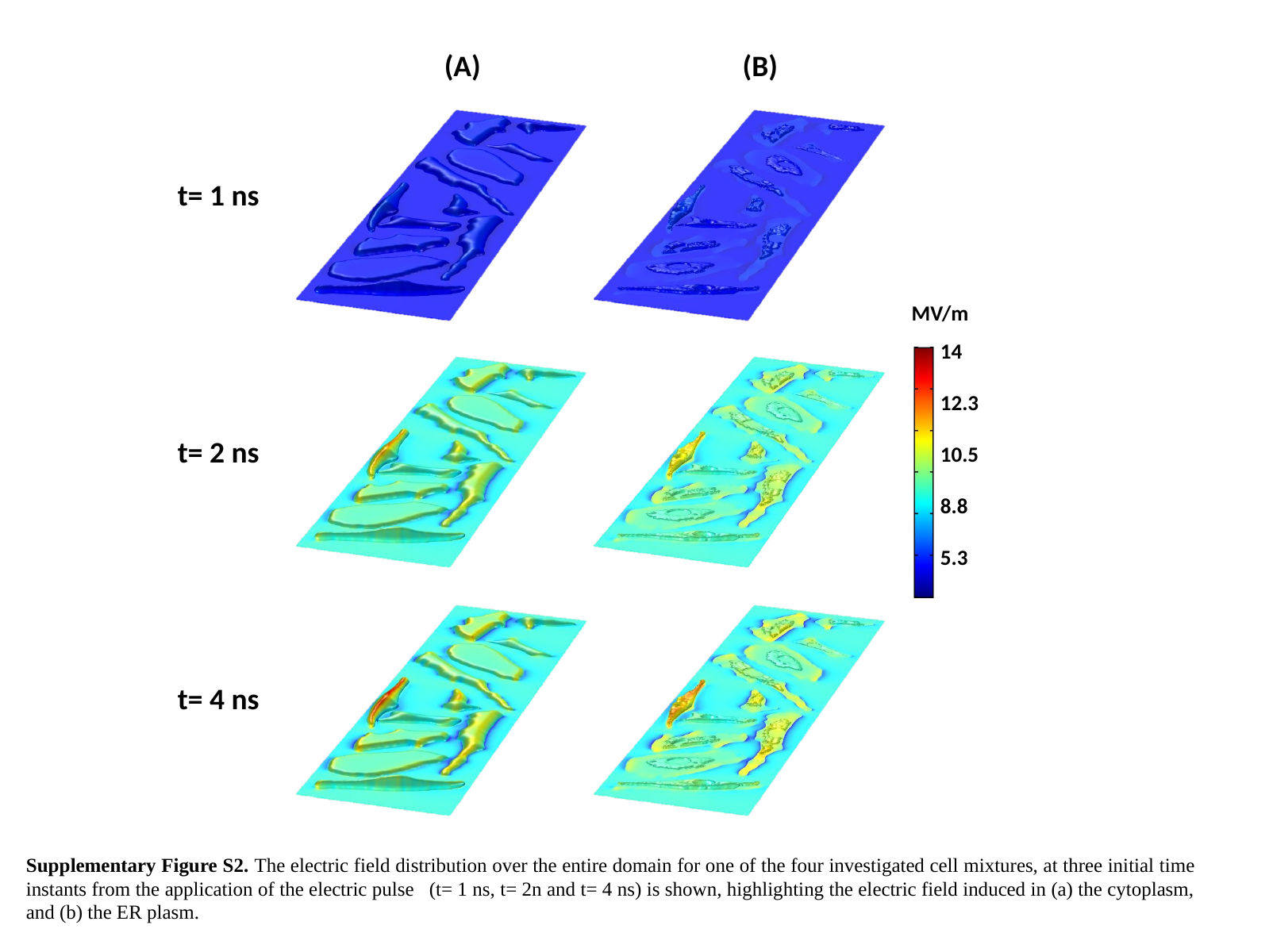

(A)
(B)
t= 1 ns
MV/m
14
12.3
10.5
8.8
5.3
t= 2 ns
t= 4 ns
Supplementary Figure S2. The electric field distribution over the entire domain for one of the four investigated cell mixtures, at three initial time instants from the application of the electric pulse (t= 1 ns, t= 2n and t= 4 ns) is shown, highlighting the electric field induced in (a) the cytoplasm, and (b) the ER plasm.

## Slide 4
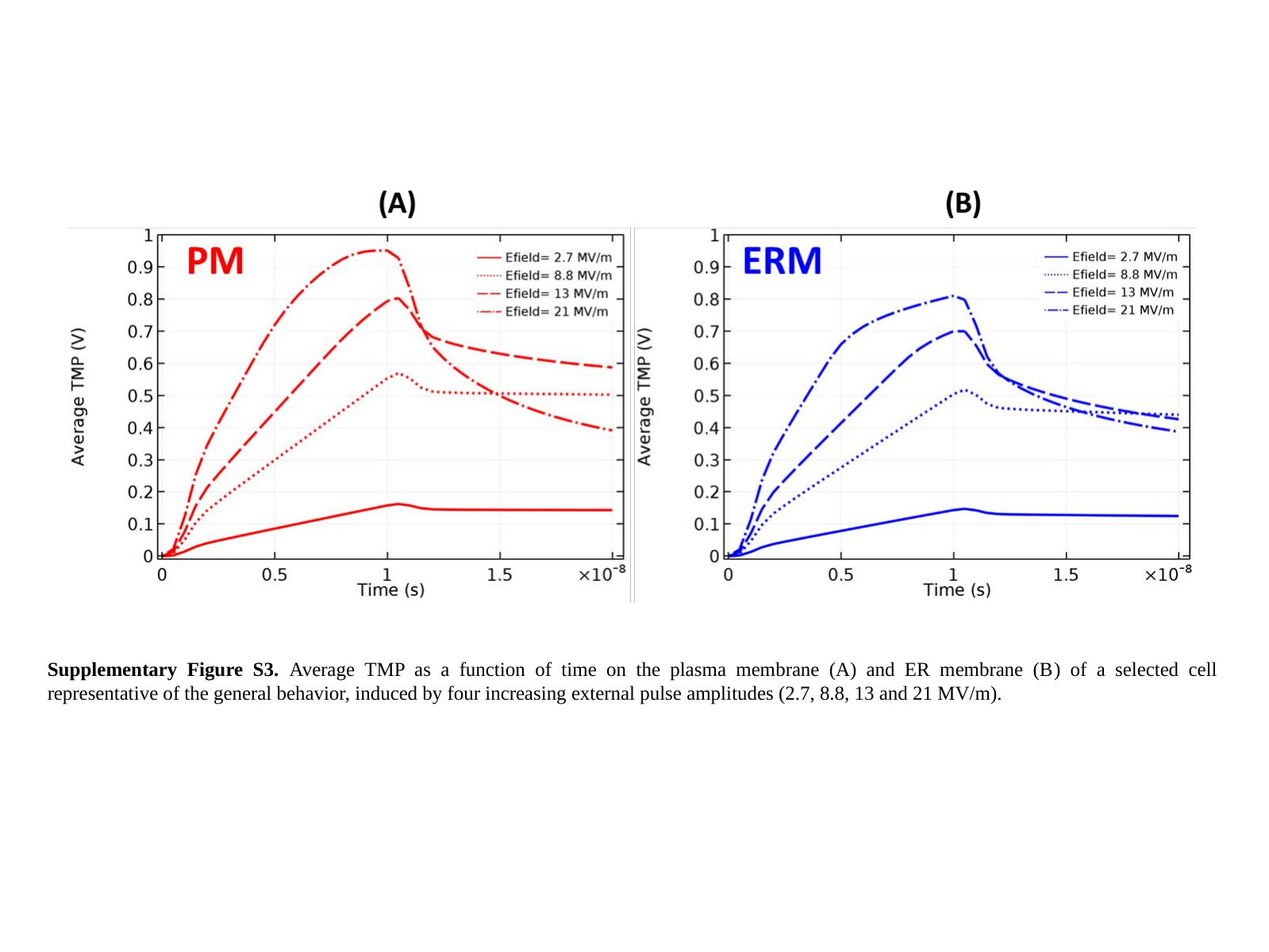

Supplementary Figure S3. Average TMP as a function of time on the plasma membrane (A) and ER membrane (B) of a selected cell representative of the general behavior, induced by four increasing external pulse amplitudes (2.7, 8.8, 13 and 21 MV/m).

## Slide 5
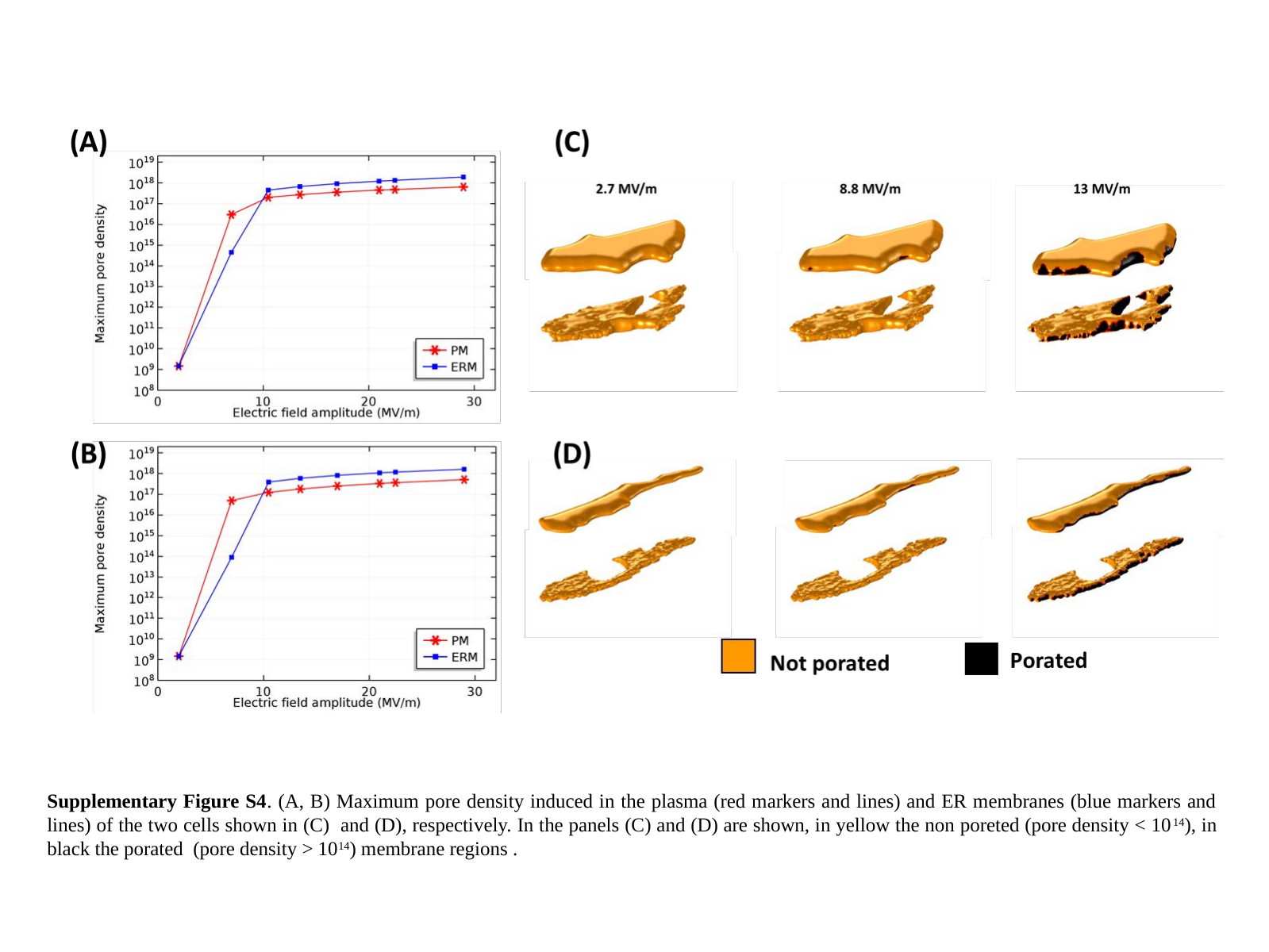

Supplementary Figure S4. (A, B) Maximum pore density induced in the plasma (red markers and lines) and ER membranes (blue markers and lines) of the two cells shown in (C) and (D), respectively. In the panels (C) and (D) are shown, in yellow the non poreted (pore density < 1014), in black the porated (pore density > 1014) membrane regions .

## Slide 6
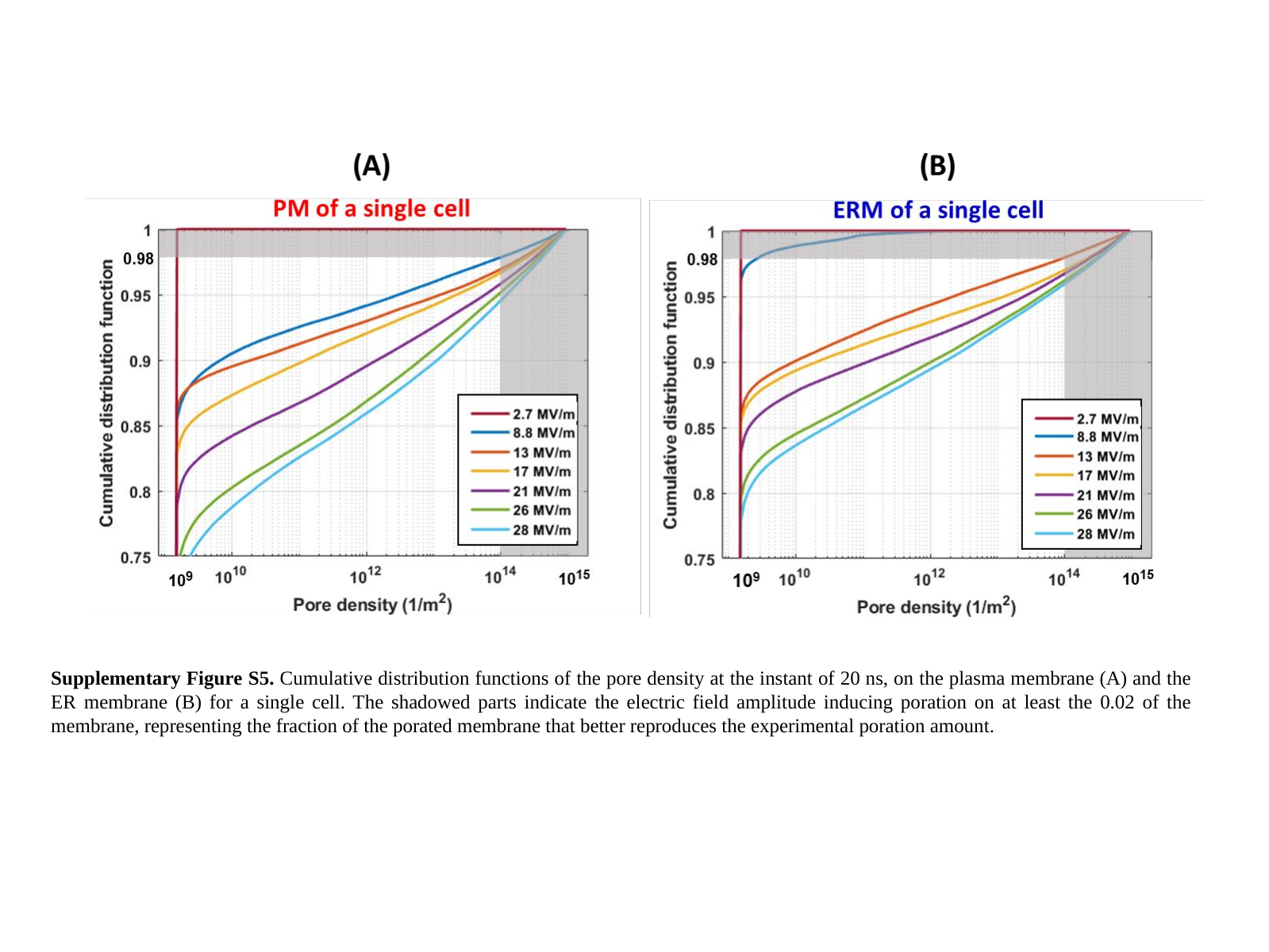

Supplementary Figure S5. Cumulative distribution functions of the pore density at the instant of 20 ns, on the plasma membrane (A) and the ER membrane (B) for a single cell. The shadowed parts indicate the electric field amplitude inducing poration on at least the 0.02 of the membrane, representing the fraction of the porated membrane that better reproduces the experimental poration amount.

## Slide 7
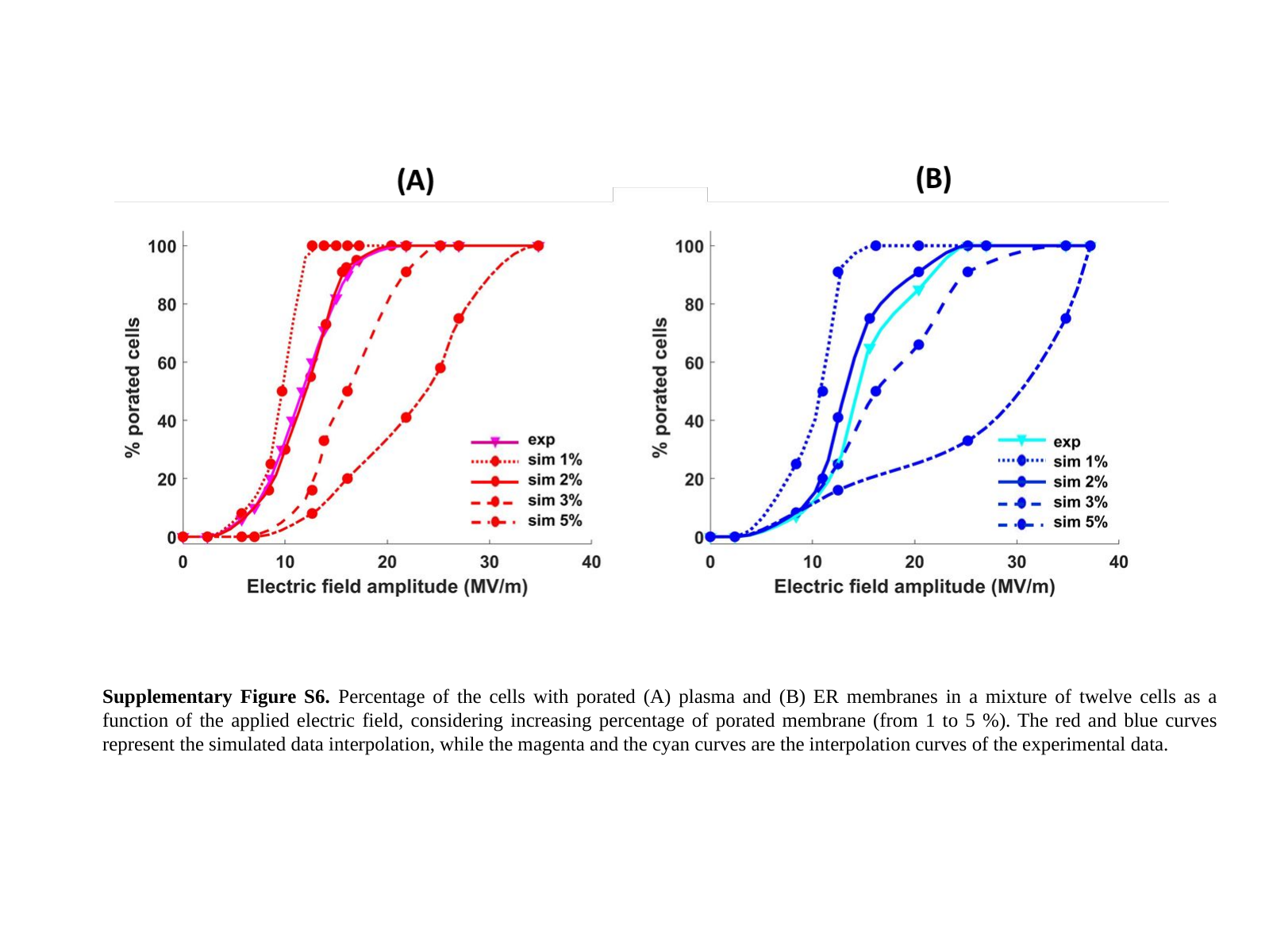

Supplementary Figure S6. Percentage of the cells with porated (A) plasma and (B) ER membranes in a mixture of twelve cells as a function of the applied electric field, considering increasing percentage of porated membrane (from 1 to 5 %). The red and blue curves represent the simulated data interpolation, while the magenta and the cyan curves are the interpolation curves of the experimental data.
